# Clinical Manifestations and Management of Prurigo in Pregnancy: A Systematic Review

**DOI:** 10.7759/cureus.98216

**Published:** 2025-12-01

**Authors:** Dana Hendie, Marwah Y Qadi, Elaf Aljohani, Maria D Alrafi, Bedoor Alghanmi, Rand Alfawaz, Reema Almasoudi, Ali Alghamdi, Relam Alhassani, Lara M Aljohani, Malak Alkhatieb

**Affiliations:** 1 Obstetrics and Gynaecology, Mohammed Bin Rashid University of Medicine and Health Sciences, Dubai, ARE; 2 Obstetrics and Gynaecology, Taibah University, Medina, SAU; 3 Obstetrics and Gynaecology, Fakeeh College for Medical Sciences, Jeddah, SAU; 4 Medicine, Almaarefa University, Diriyah, SAU; 5 Obstetrics and Gynaecology, King Abdulaziz University, Jeddah, SAU; 6 Obstetrics and Gynaecology, Qassim University, Buraydah, SAU; 7 Obstetrics and Gynaecology, Umm Al-Qura University, Makkah, SAU; 8 Obstetrics and Gynaecology, King Faisal University, Hofuf, SAU; 9 Obstetrics and Gynaecology, Almaarefa University, Diriyah, SAU; 10 Obstetrics and Gynecology, Maternity and Children Specialist Hospital, Jeddah, SAU

**Keywords:** atopic eruption of pregnancy, dermatoses of pregnancy, gestational prurigo, papular dermatitis, polymorphic eruption, prurigo of pregnancy

## Abstract

A particular type of dermatosis known as prurigo of pregnancy (PP) is typified by highly itchy papulonodules that primarily affect the extremities' extensor surfaces and the hand and feet dorsum. Despite PP not being a rare illness, there is surprisingly a distinct lack of studies exploring its depth. Therefore, this study seeks to thoroughly describe the clinical manifestation and treatment of pregnancy-related prurigo. Additionally, it aims to provide a comprehensive and up-to-date assessment of the effectiveness of different approaches to pregnancy prurigo therapy. This systematic review was conducted according to the Preferred Reporting Items for Systematic Reviews and Meta-Analyses (PRISMA) guidelines. The systematic review searched PubMed, EMBASE, Web of Science, and Cochrane databases from the databases' establishment to 13 August 2024. The studies were deemed included if they were randomized controlled trials (RCTs), cohort studies, case-control, cross-sectional, case series, and case reports; if they reported clinical manifestations and treatment for PP; and if they were written in the English language. Studies were excluded if they did not meet the aforementioned inclusion criteria; moreover, they were excluded if they were of high risk of bias or low quality. The articles included were assessed using the Newcastle-Ottawa scale and methodological quality, and the synthesis of non-randomized studies. Four different studies, with a total of 63 patients, were included in our article. The typical rash of PP is described in most patients with variable onset and distribution on the body, from the extremities to the back to the buttocks. PP can be associated with a variety of conditions, including a history of other skin conditions, such as intrahepatic cholestasis and other medical conditions. Different treatments for PP in pregnant women were reported across the included studies, most commonly a combination of topical steroids, emollients, oral antihistamines, oral corticosteroids, and ultraviolet narrow band (UVB). The effectiveness of the various therapies is variable; some patients recover before delivery, while others recover months postpartum. This systematic review emphasizes how complicated PP is in terms of both its clinical manifestations, associations, and management. The evidence points to the urgent need for a more uniform approach to diagnosis and management due to substantial variability in the clinical features, onset, and treatment responses. The study emphasizes the need for additional, larger-scale research using standardized outcome measures to identify characteristics that predict treatment success and allow for more robust comparisons of treatment modalities.

## Introduction and background

Prurigo of pregnancy (PP) is a specific dermatosis of pregnancy characterized by intensely pruritic papulonodules most commonly affecting the extensor surfaces of the arms and legs, as well as the dorsum of the hands and feet, although lesions may also appear on the trunk. Individual papules are typically small and discrete but may cluster into excoriated, crusted, or eczematous plaques due to persistent scratching [1]. PP affects approximately one in 300 to one in 450 pregnancies and usually begins during mid-gestation, around 25-30 weeks’ gestation [2,3]. Symptoms may gradually progress and can persist for several weeks postpartum [4].

Diagnosis is clinical, as no characteristic histopathologic, laboratory, or immunofluorescence abnormalities have been identified [5]. The underlying pathophysiology remains uncertain, though potential associations with pruritus gravidarum, obstetric cholestasis, and a family history of pruritic pregnancy dermatoses have been described [1,3]. Importantly, PP is distinct from other pregnancy-specific dermatoses, such as atopic eruption of pregnancy, polymorphic eruption of pregnancy, and pemphigoid gestationis, which differ in morphology, distribution, immunologic profile, and maternal-fetal risk [6,7]. Unlike these conditions, PP is considered benign and is not associated with adverse pregnancy outcomes.

Management focuses on symptomatic relief and typically includes moderate- to high-potency topical corticosteroids, pregnancy-safe oral antihistamines (e.g., diphenhydramine or chlorpheniramine), cooling measures, and menthol-based topical antipruritic preparations. In cases of severe pruritus, a short course of oral corticosteroids may be considered [1,4].

To our knowledge, this is the first systematic review to comprehensively summarize the clinical presentation, diagnostic features, and therapeutic approaches for PP. This review aims to clarify the diverse ways PP manifests in pregnant women and to provide an updated assessment of available treatment strategies and their effectiveness.

This article has been previously presented as a poster at the 2025 RCOG World Congress.

## Review

Methods

Literature Search Strategy

This systematic review was done in accordance with the Preferred Reporting Items for Systematic Reviews and Meta-Analyses (PRISMA) 2020 guidelines [6]. A systematic review search was undertaken by one researcher (MQ) in PubMed, Google Scholar, Ovid MEDLINE, and the Cochrane Central Register of Controlled Trials (CENTRAL) from the databases' establishment to 13 August 2024. The search terms were designed and input into the previously mentioned databases by one researcher (MQ) with no filters or limits. A combination of Medical Subject Heading (MeSH), such as “Prurigo”, “Prurigo of Pregnancy”, “Pruritic Eruption”, “Gestational Prurigo”, “Prurigo Gestationis”, “Polymorphic eruption", “Papular dermatitis”, “Prurigo nodularis”, “Nodular prurigo”, or “Atopic eruption of pregnancy”, and “Clinical Manifestations”, “Symptoms,” “Signs”, “Clinical Features”, “Dermatologic Manifestations,” “Skin Lesions”, “Pruritus”, or “Itching” and “Management”, “Treatment”, “Therapy”, “intervention”, or “Care” and “Pregnancy”, “Pregnant Women”, “Gestation”, “Antenatal”, or “Prenatal”. The bibliography of the selected papers was hand-searched for any additional articles.

Inclusion and Exclusion Criteria 

The literature was examined, focusing on PP in individuals aged ≥18 years. The studies were deemed eligible if they were (1) randomized controlled trials (RCTs), cohort studies, case-control, cross-sectional, case series, and case reports; (2) they reported clinical manifestations and treatment for PP; (3) they were written in the English language. Studies were excluded if they did not meet the aforementioned inclusion criteria; moreover, they were excluded if they were of high risk of bias or low quality based on the assessment of study design, sample size, data collection and analysis, and other relevant variables.

Selection of Articles and Data Extraction

Four authors (MA, RA, RA, and LA) independently evaluated the titles and abstracts of publications for the complete text reading using Rayyan (Rayyan Systems Inc., Cambridge, MA) after removing duplicates. Then, five authors (DH, LA, AA, RA, and EA) additionally independently examined each article's text in its entirety. Any disagreements were resolved by one researcher (MQ).

Three writers (MA, RA, and BA) independently retrieved the subsequent details from each eligible paper after distributing the articles that were selected among them: title of the article, year of publication, name of the journal, country of origin, and study design, as well as details about the patients participating, the total number of patients, age, and number of previous pregnancies. The following information about prurigo and previous medical history was also recorded: gestational week at prurigo onset, duration of the illness, lesion distribution, pruritus and eruption in obstetric history, and any other significant medical history. Moreover, details about the treatment were taken, including its type, name, dosage, and response to treatment. Treatment effectiveness and effect, safety, and adverse effects were also documented.

Quality Assessment 

Two researchers (DH and EA) evaluated the research quality using the Newcastle-Ottawa scale (NOS) modified to be used to evaluate cross-sectional studies. The NOS was created to evaluate the quality of nonrandomized studies, and it utilizes a "star system" to assess articles in three general aspects: the choice of study groups, the comparability of the study groups, and the determination of either the exposure or the outcome of interest [7,8]. The studies were designated to be of either “good”, “fair”, or “poor” quality described previously by McPheeters et al. [9].

The methodological quality and synthesis of case series and case reports used to evaluate case series and case reports have four domains: selection, ascertainment, causation, and reporting. When developing this tool, Murad et al. stated that it would be preferable to identify the questions that are most crucial to the study and evaluate the chosen publications based on those questions rather than assigning a numerical score to each reviewed study [10]. We decided that the most vital aspect is a clear description of the illness and treatment, in addition to describing the response to this treatment. Studies were described as having good, adequate, or poor quality reporting based on their answers to the aforementioned questions. The research papers were evaluated independently, and any discrepancies were resolved by discussion.

Results

Study Selection 

**Figure 1:**
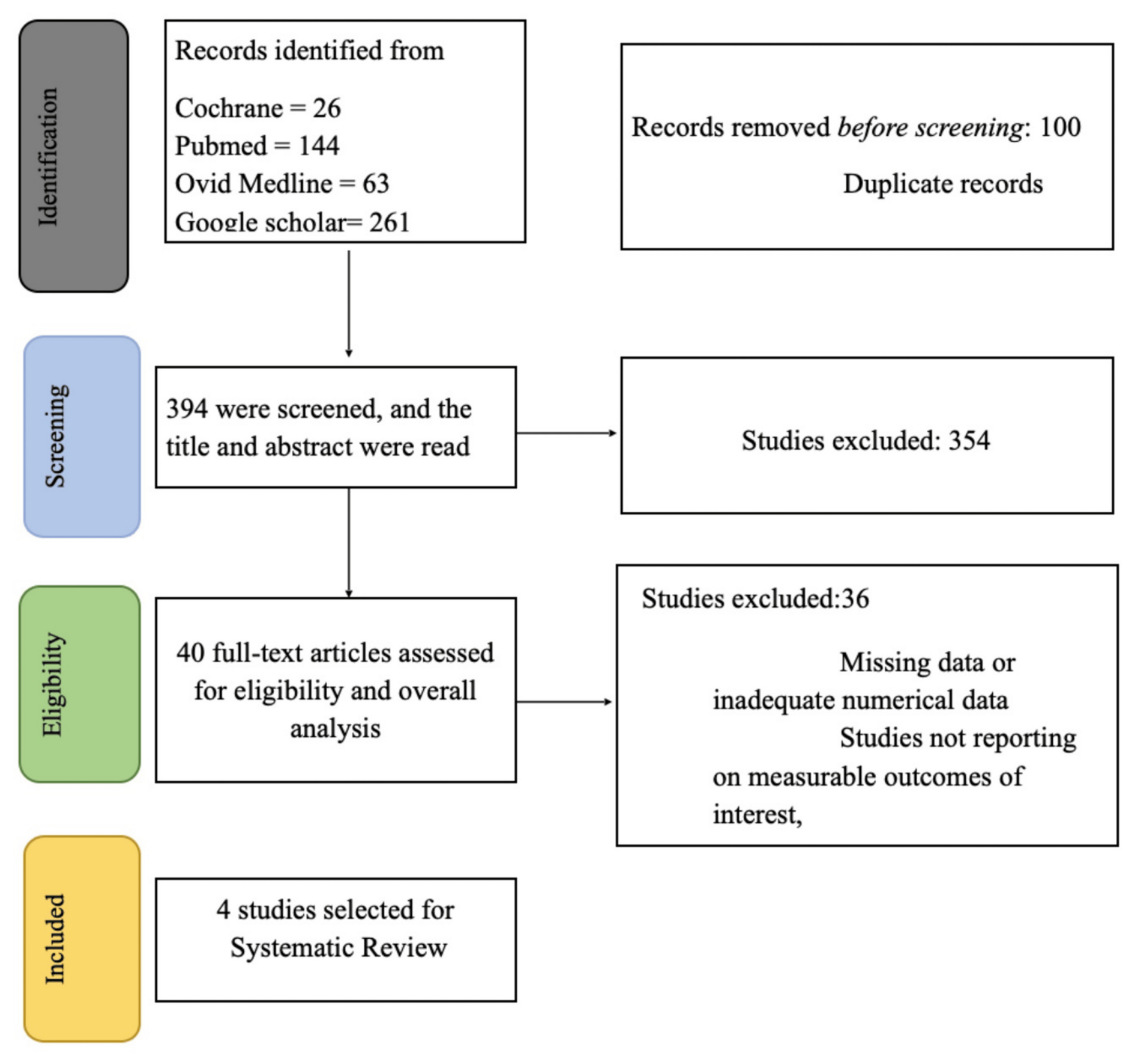
PRISMA diagram PRISMA: Preferred Reporting Items for Systematic Reviews and Meta-Analyses

A database search was performed, and 494 articles were retrieved for screening. Two independent authors (AS and BS) carried out the screening in two stages. After eliminating duplicates, 349 studies remained. The first stage is the title and abstract screening, and 354 were removed due to studies being out of the scope of the current research. After the first stage, the number of articles remaining was 40. Full-text screening was performed on the remaining studies, and 36 studies were excluded. The final four articles were included in our article (Figure 1).

Study Characteristics 

Four studies were incorporated in total. The research comprised two case series, a single case report, and one cross-sectional study. Among the 311 patients, 63 had PP, with an average age ranging from 21 to 43 years [3] and 15 to 44 years [2]. The individuals came from the United States, Brazil, Australia, Egypt, and Germany. PP appeared between 14 and 33 weeks of gestation [3], specifically between 26 and 28 weeks [11], with occurrences in three patients under 20 weeks, seven patients from 20-24 weeks, 10 patients from 25-29 weeks, six patients from 30-34 weeks, six patients from 35-39 weeks, and seven patients over 40 weeks [2]. In total, there were 29 primigravida patients and 22 multigravida patients. The diagnosis of PP was established when typical features of PP (excoriated pruritic erythematous papulonodules) were observed without an atopic background [3], alongside normal liver function and bile acid levels [11] and a histological assessment consistent with PP [12], or primarily papular lesions accompanied by excoriation. In certain instances, the rash is characterized by maculopapular erythema or linked to secondary eczematous changes resembling discoid eczema [2]. The distribution of lesions in PP typically occurs in the abdomen and extremities in many studies, but it can also extend to the shoulders, back, and buttocks [3]. In addition, PP typically lasts from three weeks to 21 weeks [3]. PP may be associated with various conditions, including a background of other skin disorders such as polymorphic eruption of pregnancy, gestational pruritus, prurigo nodularis, and intrahepatic cholestasis during pregnancy. Additionally, it may be linked to hypothyroidism, anemia, mental health issues, allergies, bariatric surgery, and obesity (Table 1).

**Table 1:** Characteristics of the included studies NR: not reported

Author, year	Country	Study Design	Sample Size	Patients with Prurigo	Age (Yrs)	Parity	Gestation Week	Lesion description	Lesion Distribution	Prurigo of pregnancy duration weeks	Medical history
Ravelli, 2020 [3]	United States, Brazil, and Europe	case series	20	20	21–43	Primigravida= 8 Multigravida=12	14-33 weeks (mean gestational age: 24.8 weeks; median gestational age: 27.5 weeks)	Excoriated, erythematous papules	Abdomen, shoulders, back, chest, all extremities, upper extremities, and buttocks	3-21 Weeks	Polymorphic eruption of pregnancy n = 2 (16.6%) prurigo of pregnancy (n = 2 (16.6%). Gestational pruritus (n = 1 (8.3%). Pruritic skin conditions (prurigo nodularis and pruritus of unclear etiology) N=2 (10%). Psychiatric disorder N=4 (20%). Hypothyroidism N=2 (10%). Obesity N=2 (10%). Diabetes N=1, Allergies N=1, Seasonal allergies N=1
Nurse, 1968 [2]	Australia	Case series	40	40	15-44	Primigravida=20 Multigravida=20	< 20 weeks = 3 20-24 Weeks = 7 25-29 Weeks = 10 30-34 Weeks = 6 35-39 Weeks = 6 ≥ 40 Weeks = 7 after delivery = 1	Papular lesions with excoriation, maculopapular erythema	Abdomen and limbs	NR	Anemia N=1, History of pruritus in previous pregnancies N=2
Kaur, 2013 [11]	Egypt	Cross sectional study	250	2	NR	NR	26th and 28th week	Discrete excoriated papules and nodules	Limbs and abdomen	NR	NR
Müller, 2023 [12]	Germany	Case report	1	1	3	Primigravida	NR	Multiple excoriated papules and nodules	Trunk and limbs	NR	Genetic predisposition to cholestatic liver disease, history of intrahepatic cholestasis of pregnancy, bariatric surgery, and obesity

Management and Treatment for PP

Various treatments and management strategies for PP were noted across the studies reviewed. For instance, Ravelli et al. [3] indicated that PP was managed using oral antihistamines, topical steroids, augmented betamethasone, prednisone, emollients, benzoyl peroxide, and narrowband ultraviolet light B. Nurse [2] reported 40 cases of PP. It categorized the cases as early and late. The early type typically presents during the second trimester of pregnancy, whereas the late type emerges close to term. In both early and late types of PP, the treatment was alike. PP was typically managed with 1% phenol in oily calamine lotion and oral trimeprazine at 10 mg doses, taken two to three times a day or at night. In contrast, in some instances, barbiturates such as quinalbarbitone 200 mg at night were effective. In instances of secondary eczematous alterations, topical steroids, trimeprazine, and erythromycin were required for complete recovery. Kaur et al. [11] documented two instances of PP, where both patients received topical steroids and oral antihistamines. The study by Müller et al. [12] indicates that chronic nodular prurigo, previously managed with topical steroids, high-dose oral non-sedating antihistamines, oral prednisolone, gabapentin, azathioprine, and ursodeoxycholic acid without success, can be effectively treated using 0.02 mg/kg naloxone. The aforementioned information is visualized in Table 2.

**Table 2:** Treatment of prurigo NR: not reported

Author, Year	Treatment for Prurigo in pregnancy		
	Type of treatment (class)	Dosage	Response to treatment
Ravelli, 2020 [3]	Oral antihistamines, topical steroids, augmented betamethasone, prednisone emollients, benzoyl peroxide, barrowband ultraviolet light B	Prednisone at a dose of 20-30 mg/day	N=5 partial response to treatment or resolved at or immediately after delivery, N=15 satisfactory response to treatment and faded prior to delivery, N=6 treated with oral prednisone due to lack of response with other modes of treatment, N=1 treated well with narrowband ultraviolet light B
Nurse, 1986 [2]	Phenol in oily calamine lotion and trimeprazine, sedatives like barbiturates, steroid creams, prednisone, oral chlorpromazine, erythromycin	Trimeprazine 10 mg three times daily,1% phenol, 200 mg of quinalbarbitone	Early type = at the time of delivery, Late type = 3rd-4th week postpartum, Associated with secondary eczematous changes = 3 months
Kaur, 2013 [11]	Topical steroids and systemic antihistamines	NR	Spontaneous resolution of lesions within 4 weeks postpartum
Muller, 2002 [12]	Topical steroids: High-dose oral non-sedating antihistamines, oral prednisolone, gabapentin, azathioprine, ursodeoxycholic acid, naloxone	Gabapentin 900 mg daily, azathioprine 150 mg daily, naloxone 0.02 mg/kg	Treatment response to naloxone within 4 weeks

Outcome Measure and Findings 

Ravelli et al. [3] noted that a total of 15 patients attained complete recovery with treatment, and the rash disappeared prior to delivery; five patients showed a partial response to treatment or recovery at or after birth, while six patients needed oral corticosteroids due to an inadequate response to other therapies, and one patient was successfully treated with narrowband ultraviolet light B. Nurse [2] recorded that, in the early type, the rash often clears by the delivery date; however, in the late type, it usually resolves during the third to fourth week after childbirth. However, if secondary eczematous alterations complicate PP, the rash might require up to three months to resolve. Kaur et al. [11] noted that natural resolution occurred in two patients within four weeks after giving birth. Furthermore, Müller et al. [12] stated that treatment with naloxone cleared the rash in four weeks (Table 2).

Quality Assessment

One study [11] was evaluated by the NOS tool, and it was found to be of a fairly adequate quality (Table 3). The methodological quality and synthesis of case series and case reports were assessed in three publications. Nurse [2] and Müller et al. [12] were regarded as being of high quality. In contrast, Ravelli et al.'s [3] study was deemed to have adequate reporting quality (see Table 4 for further details).

**Table 3:** Newcastle–Ottawa quality assessment scale (NOS) modified for cross-sectional studies

Authors (Year)	Selection				Comparability	Outcome		Final score
	Representativen ess of the sample	Sample size	Ascertainment of exposure	Non-respondents	Comparability of outcome groups	Assessment of outcome	Statistical test	
Kaur, 2013 [11]	*	*	*	-	**	**	-	7

**Table 4:** Evaluating the methodological quality of case reports and case series Selection: (Q. 1). Does the patient(s) represent(s) the whole experience of the investigator (center) or is the selection method unclear to the extent that other patients with similar presentations may not have been reported? Ascertainment: (Q. 2). Was the exposure adequately ascertained? (Q. 3). Was the outcome adequately ascertained? Causality: (Q. 4). Were other alternative causes that may explain the observation ruled out? (Q. 5). Was there a challenge/rechallenge phenomenon? [question 6]. Was there a dose-response effect? (Q. 7). Was follow-up long enough for outcomes to occur? Reporting: (Q. 8) Is the case(s) described with sufficient details to allow other investigators to replicate the research or to allow practitioners to make inferences related to their own practice?

Reference	Selection	Ascertainment		Causality		Reporting			Overall Score
Questions	Q. 1	Q. 2	Q. 3	Q. 4	Q. 5	Q. 6	Q. 7	Q. 8	
Ravelli, 2020 [3]	YES	YES	YES	NO	NO	NO	NO	YES	4
Nurse, 1968 [2]	YES	YES	YES	NO	NO	YES	YES	YES	6
Müller, 2023 [12]	YES	YES	YES	YES	NO	NO	YES	YES	6

Discussion

PP was first named prurigo gestationis; it was described by Besnier et al. in 1904, where they classified all dermatoses lacking blisters under this definition [13,14]. One of our included studies by a nurse in 1968 classified PP into two different types: early and late-onset prurigo. Early onset was described as manifesting in the middle trimester with characteristic features of PP. Late-onset begins near term and has a mixture of erythematous multiform features and typical PP that also includes cases of polymorphic eruption of pregnancy (PUPP) [5,15]. Therefore, in our present day, the term "prurigo of pregnancy” includes Besnier PP, Nurse’s early onset prurigo, and Spangler papular dermatitis of pregnancy. PP is not a rare occurrence, as it was described to occur in one in 300 to one in 450 pregnancies. However, some studies from India have reported an even higher incidence, surpassing that of PUPP [2,3].

PP typically starts during mid-pregnancy, around 25-30 weeks of gestation, although onset in other trimesters has been documented [1,3,15]. A scarce amount of information is known about the pathophysiology of PP. According to previous studies, most PP patients had a relationship with atopy [15]. However, as cases without atopic backgrounds have been documented, the link to atopy is still debated [3]. Some postulate that PP is associated with a family intrahepatic cholestasis (ICP) history. However, it can be differentiated by the different characteristic lesions [16]. PP lesion is usually described as exceedingly pruritic individual papulonodules distributed over the hands and feet dorsum and the extremities' extensor surface. It can also be located in other sites, such as the abdomen, buttocks, and shoulders [1,3]. The disease usually resolves after delivery, but can persist for up to three months postpartum. The diagnosis of PP is clinical; laboratory tests are usually normal, and a skin biopsy is not done routinely. If a skin biopsy is done, it reveals persistent chronic inflammatory cell infiltration in the upper dermis and acanthosis with parakeratosis due to excoriations [4,16]. Treatment of PP, often symptomatic, moderately and highly potent topical steroids have a beneficial effect on the lesions. Other medications, such as oral antihistamines (e.g., chlorpheniramine and diphenhydramine), can also be utilized [1,16]. Symptomatic treatment, such as antipruritic topical creams containing menthol and cooling baths, can also aid in the patient's recovery. Oral corticosteroids can be used in cases of severe pruritus [1]. Moreover, some patients with PP have greatly benefited from narrow UVB treatments [16].

Despite the challenges inherent in conducting research on pregnancy-related dermatological conditions, this review successfully compiled data from a range of study designs, including case reports and case series, and cross-sectional studies, encompassing a geographically diverse patient population from the United States, Brazil, Australia, Egypt, and Germany. This broad representation enhances the generalizability of the findings, though further research across a wider range of geographical locations is still warranted. Furthermore, there is a distinct lack of articles with higher evidence study designs such as systematic reviews, cohort studies, and case control studies. Therefore, further research with a wide array of study designs should be conducted in the future.

The analysis of the included studies revealed considerable heterogeneity in the clinical presentation of PP. While severe pruritus and characteristic skin changes formed the core diagnostic criteria, the gestational age at onset varied significantly, ranging from early pregnancy to the postpartum period. The distribution of the lesion is also quite variable; while mostly afflicting the extremities, it can also affect the back, buttocks, and shoulders. The presence or absence of a history of atopy and the potential role of other diseases, such as diabetes and IC,P further complicated the diagnostic picture, highlighting the need for a more nuanced understanding of the condition's pathophysiology. The observation that PP can manifest in individuals without a pre-existing atopic background challenges existing assumptions and suggests a more complex etiology than previously understood. Future research should focus on identifying specific biomarkers, genetic predispositions, or illnesses that might contribute to the development of PP.

The treatment strategies employed in the included studies were equally diverse, reflecting the current lack of established treatment guidelines. The range of therapeutic approaches, from topical steroids and emollients to systemic antihistamines, prednisone, and even UVB phototherapy, underscores the need for a more standardized and evidence-based approach. The varied treatment responses were observed, with some patients experiencing complete resolution within 24 hours, while others showed spontaneous resolution only weeks postpartum, further emphasizing the heterogeneity of the condition and the need for personalized treatment strategies. Further research is needed to fully understand how prurigo affects the skin's long-term health, including investigating the lasting impact seen after lesions have healed.

A significant limitation of this review lies in the relatively small number of included studies and the inherent limitations of the study designs used. The retrospective nature of some studies introduces potential biases related to recall and data collection. Furthermore, the lack of standardized outcome measures across studies hinders direct comparison of treatment efficacy. Future research should prioritize larger-scale, prospective studies with standardized outcome measures to allow for more robust treatment modality comparisons and to identify treatment response predictors. Such studies should also incorporate detailed information on patient demographics, medical history, and pregnancy-related factors to better understand the interplay of these variables in the development and progression of PP. Developing a standardized diagnostic tool and a comprehensive treatment algorithm based on robust evidence would significantly improve patient care and reduce the burden of this distressing condition. Integrating advanced imaging techniques and genetic analysis could provide valuable insights into the underlying pathophysiological mechanisms. It is also vital to note that the inclusion of Nurse's 1986 study [2] can cause heterogeneity in the results as he classified PP into an early and late type, with the late type being a mix of typical PP and erythematous multi-forme, later reclassified as PUPP [2].

## Conclusions

This systematic review highlights the complexity of prurigo during pregnancy, both in its clinical features and treatment. While the clinical features, onset, and treatment responses demonstrate considerable variability, evidence indicates a pressing need for a more standardized approach to diagnosis and management. It underscores the necessity for personalized treatment plans customized to individual patient profiles. The study highlights the need for a larger scale. These prospective studies utilize standardized outcome measures to facilitate more substantial comparisons of treatment methods and identify factors that predict treatment effectiveness.
